# Pharmacophore-Based QSAR Model of Multi Scaffolds as NAMPT Inhibitors & Scaffold Diversity Analysis

**DOI:** 10.3390/molecules31101773

**Published:** 2026-05-21

**Authors:** Sujin Lee, Mei Zheng, Kang Kim, Kwang-Hoon Chun

**Affiliations:** 1 College of Pharmacy, Daegu Catholic University, Gyeongsan 38430, Republic of Korea; 2 Gachon Institute of Pharmaceutical Sciences, College of Pharmacy, Gachon University, Incheon 21936, Republic of Korea

**Keywords:** NAMPT, receptor-guided pharmacophore model, pharmacophore based QSAR model, shape-based similarity

## Abstract

NAD+ plays crucial roles in various biological processe and its aberrant regulation has been suggested to be critical in the pathogenesis of diverse diseases. Intracellular NAD+ is synthesized largely from nicotinamide mononucleotide (NMN), which is the product of reaction catalyzed by nicotinamide phosphoribosyltransferase (NAMPT). Thus, the development of specific inhibitors targeting NAMPT has been suggested as a promising treatment strategy. In this study, we developed a pharmacophore-based QSAR model to discover novel NAMPT inhibitors based on diverse structural features. By virtual screening using the conformation model, we could identify eight novel active analogs having distinct pharmacophores. The biological activity of these candidates on cell viability were further examined. Our study proves the efficiency of our novel screening model and demonstrates its usefulness in the application of drug discovery process.

## 1. Introduction

Cancer cells require higher NAD+ utilization than normal cells to maintain altered metabolic needs [1]. There are several major NAD+ production pathways: the de novo pathway, the Preiss–Handler pathway, and the salvage pathway [2]. Among these, the salvage pathway is a key contributor to cancer cell proliferation, in which nicotinamide is recycled to regenerate NAD+. In this process, nicotinamide phosphoribosyltransferase (NAMPT) converts nicotinamide and 5′-phosphoribosyl-1-pyrophosphate into nicotinamide mononucleotide (NMN), a major precursor of nicotinamide adenine dinucleotide (NAD+) biosynthesis. NAMPT plays a key role as a rate-limiting enzyme in the regulating of NAD+ levels [3]. In addition to its role in NAD+ biology, NAMPT has been reported to function as an adipocytokine (named pre-B-cell colony-enhancing factor, PBEF) and as an insulin-mimetic hormone (named visfatin), suggesting a role as a potential inflammatory regulator [4].

NAD+ plays a well-established role in maintaining altered energy metabolism in cancer cells. Numerous studies have shown that NAMPT is highly expressed and play a key role as an oncogenic protein in the progression of various cancers, including colorectal cancer [5,6], breast cancer [7], osteosarcoma [8], and so on. NAMPT inhibitors exhibit diverse effects on glycolysis, oxidative phosphorylation, amino acid metabolism, the pentose phosphate pathway, nucleotide metabolism, and lipid metabolism [2]. In addition to the role in energy metabolism, NAMPT has been suggested to be involved in sirtuin function, DNA repair system, redox balancing, and epithelial–mesenchymal transformation (reviewed in [2]). Collectively, these findings suggest that NAMPT is an attractive therapeutic target in cancer treatment.

NAMPT has been considered a plausible therapeutic target for managing aberrant NAD+ production in clinical settings. Furthermore, combination therapies with other anticancer agents are being evaluated in various preclinical models. The development of next-generation NAMPT inhibitors with improved selectivity and efficacy, along with fewer off-target effects, warrants novel approaches based on rational drug design.

Notably, many NAMPT regulators in clinical trial or in clinical use have been developed based on a nicotinamide (NAM) scaffold. For example, the two most prominent NAMPT inhibitors, FK866 (APO866) and GMX1777 (a prodrug of GMX1778), have clinical indications against cutaneous T-cell lymphoma and B-cell chronic lymphocytic leukemia (APO866), as well as metastatic melanoma (GMX1777), in clinical trials [9]. Although efforts have been made to identify inhibitors with novel scaffolds though high-throughput screening, the resulting lead compounds have largely been based on common structures, including a cap group mimicking the pyridine moiety of nicotinamide, linker, and tail group [10,11,12,13]. Therefore, the development of NAMPT inhibitors with truly novel structures, requires the application of distinct strategies based on new design frameworks.

In this study, we investigated the structural diversity of NAMPT ligands in chemical space using novel quantitative structure–activity relationship (QSAR) models. To this end, we [1] constructed pharmacophore models along with corresponding 3D-QSAR models based on previously reported multiple scaffolds of NAMPT inhibitors, [2] identified new fragment-sized hit compounds through high-throughput virtual screening using the validated models, and [3] evaluated the biological efficacy of selected in silico hit compounds identified through clustering analysis.

## 2. Results and Discussion

### 2.1. QSAR Prediction

As shown in Table 1, pharmacophore models were generated using the partial least squares (PLS) factor 1, satisfying the criteria described in the Material and Methods section. We obtained several statistical parameters, including R^2^, Q^2^, predictive R^2^, and Pearson correlation. The data showed that all models exhibited R^2^ and stability values above 0.7 and 0.9, respectively (Tables S2 and S3), suggesting a stable and reliable correlation between the predicted and experimental data.

**Figure 1:**
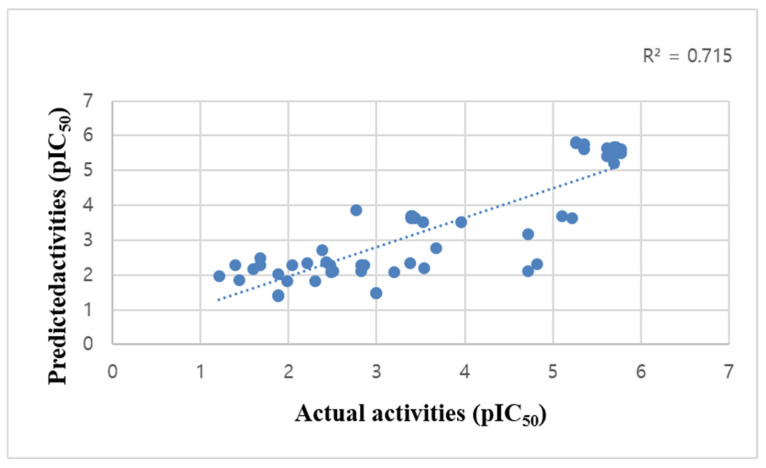
Multiple linear regression curves showing linear relationship between values of actual and predicted pIC_50_ calculated by derived QSAR model.

In particular, the model ADDRRR.146 presented the highest R^2^ and Q^2^ values, 0.85 and 0.67 respectively, and was accordingly selected as the best model. Notably, although the dataset (n = 5) comprises heterogenous chemical scaffolds (Table S1), the high Q^2^ value and statistical stability indicate that the model effectively captures essential 3D pharmacophore features that transcend specific structural classes. This chemical diversity in the training set reinforces the model’s reliability and ensures a broad applicability domain, enabling the successful identification of novel scaffolds. The linear relationship between the predicted and the measured activities on ADDRRR.146 is shown in Figure 1 and detailed in Table S1, further confirming its predictive accuracy.

### 2.2. Comparative Analysis of Pharmacophore Models with Docking Mode

**Figure 2:**
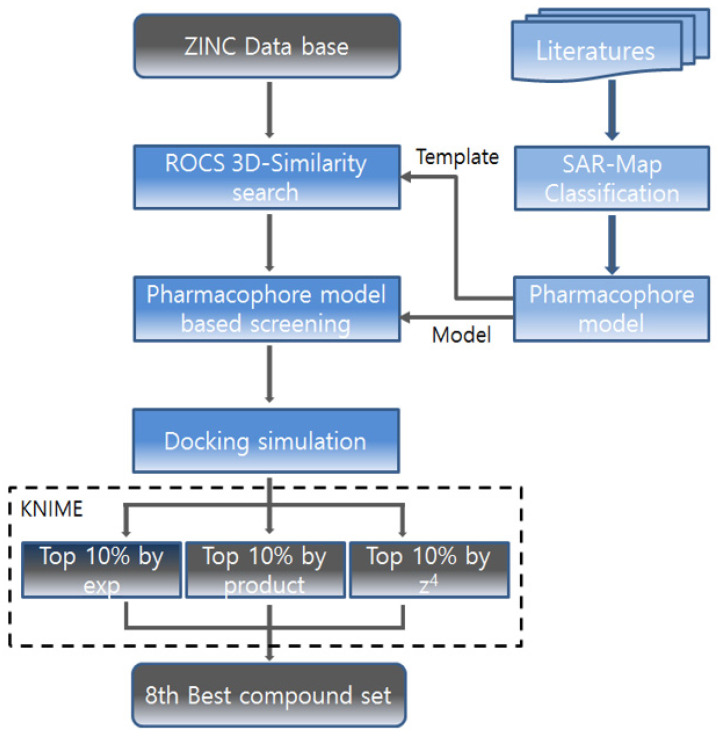
The overall work flow of screening process.

The ADDRRR.146 model closely aligned with the reported NAMPT inhibitors in three key units: cap, linker, and tail group [11]. The docking mode to the model of compound **1_14**, a previously reported active compound having 0.054 μM IC_50_ [15], is illustrated in Figure 2. These features could explain the interactions of a NAMPT inhibitor to the residues of NAMPT.

In particular, inhibitors with higher activity presented higher fitness values. The detailed interactions are as follows: First, the D7 feature of the cap region formed a hydrogen bond with PHE193 residue, and rings of R10 were sandwiched between ASP219 and TYR18 residues and formed a π–π stacking interaction. The R8 also formed a π–cation interaction with ARG196 residue. The amide (D6 feature) in the connecting unit region formed a hydrogen bond interaction with ASP219 residue. Further, it is worth noting that the phenyl group (9R9 feature) formed a π–π stacking interaction with HIS191 residue. In contrast to the previously reported analogs such as FK866 [3] and GMX1778 that applied an alkyl linker to connect to the tail group, our model introduced the R9 ring structure. This ring formed a π–π stacking interaction with HIS191 residue, and it is expected to form an additional binding interaction to target the protein. In the tail group, the A3 feature formed a hydrogen bond with a water molecule, but this is not a significant factor, and it seems to be the limit of the dataset. Finally, the terminal ring is expected to have the ability to anchor the molecule because of the interaction of the phenyl group with a hydrophobic cleft formed by near residues (Figure 2) [16]. It is possible for the A3 feature to be assigned due to chemical linkage between the tail group and the hydrophobic cleft.

### 2.3. Screening of Focused Library

**Figure 3:**
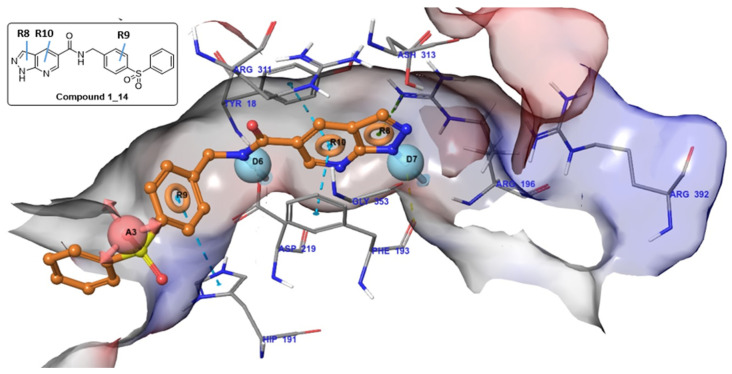
Structure of phase model ADDRRR146 and docking mode of **1_14** compound (PDB code 4M6Q). Each dotted line means interaction between phase model and protein. Yellow is hydrogen bond, sky blue is π–π stacking, and dark green is π π–cation interaction.

The overall workflow for the screening is described in Figure 3. To identify novel structures of NAMPT ligands through the in silico screening, the ZINC database was used as a compound collection. The template molecules of the ADDRRR.146 model were used as a query representing unique shape and 3D vectored features (pharmacophores) of NAMPT inhibitors. For maximum alignment and best fitness between the query (reference) and database, a large number of conformer ensembles were within the energy window of 30 Kcal/mol. Then a 3D similarity search was performed between conformers of Zinc (M) and conformers of the query molecule (N), producing 3800 similar hit compounds. ROCS (Rapid Overlay of Chemical Structures) was chosen for the ‘M by N’ 3D similarity search under efficient parallel thread processing (open MPI, OEMPI run) [16,17,18,19]. During the screening, shape-based superposition of every conformer pair followed the procedure according to (1) selection of starting position for the superposition, (2) finding ‘centers-of-mass’ of the query conformer, and (3) single selection among multiple overlapped results. Among twelve scoring values acquired from three distance metrics and weighting, the combined Tanimoto score of shape similarity with 3D vectored feature (pharmacophore) similarity was chosen as the first criterion. This initial pharmacophore-based filtering step served to rapidly narrow the chemical space by retaining only compounds whose 3D shape and feature distributions were geometrically consistent with the query, thereby enriching the candidate pool with structurally relevant scaffolds.

**Figure 4:**
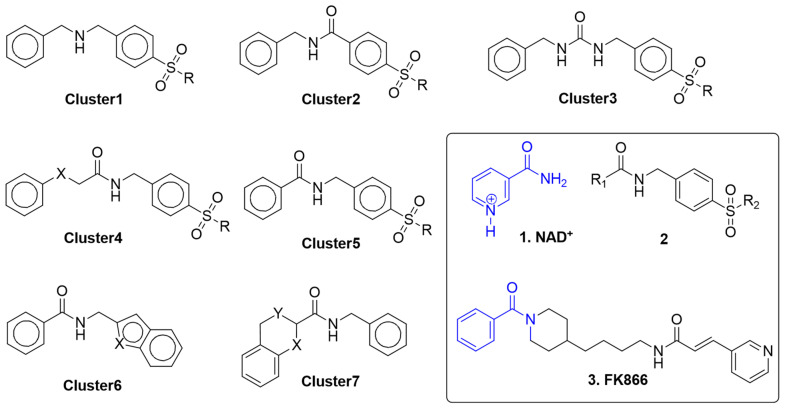
Structures of clustering.

The second 3D-similarity calculation was performed with the exact ADDRRR.146 model under PHASE to reduce 100 in silico hit compounds from 3800 compounds. This secondary pharmacophore screen applied a more stringent, model-specific fitness criterion, refining the selection to those that not only resembled the query shape but also satisfied the spatial arrangement of key pharmacophoric features critical for target engagement. With two 3D-similarity calculations, docking scoring was assigned to all 3800 compounds. Therefore, the three scores, combined Tanimoto scores (first screening: shape and pharmacophore), fitness scores (second screening: pharmacophore), predicted activity (second screening: pharmacophore and 3D-QSAR), and docking scores (third screening: structure-based screening on five protein conformers) were collected and manipulated under KNIME. Conclusively, each filtering step contributed complementary results: the pharmacophore steps provided rapid, geometry-driven enrichment, the QSAR prediction offered a quantitative estimate of potency, and the docking scores supplied binding mode-level discrimination. Together, these three stages progressively reduced the risk of advancing inactive or promiscuous compounds. The data fusion of three scores was conducted to produce Z4 (sum), Product (multiplication), and Exp (exponential function). These three fused scores became thresholds to extract only the upper 10% of scored hit compounds. The enrichment factor at 10% of the database is a standard metric used to evaluate virtual screening performance [20]. Additionally, in this study, we applied a 10% cutoff to 3800 compounds and the resulting compounds are 380, which was a tractable number for subsequent K-means clustering (Figure 4).

**Figure 5:**
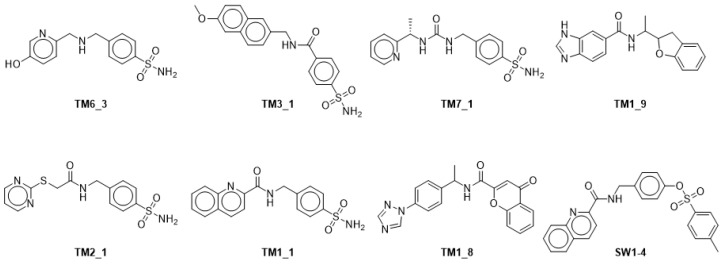
Structures of clustering. After the verification on chemical structures within each cluster, 8 compounds for bioassay finally were chosen according to the verified grouping and commercial availability.

After the verification on chemical structures within each cluster, eight compounds were finally chosen for bioassay according to their verified grouping and commercial availability (Figure 5). Compounds **TM6_3**, **TM3_1**, **TM2_1**, **TM1_1**, and **TM7_1** derived from ZINC data and **TM1_8**, **TM1_9**, and **SW1_4** were derived from the ENAMINE library.

Reported NAMPT inhibitors contain NAD^+^ (1) mimic functional groups such as pyridine or benzenesulfonamides. Based on the clusters, we identified two analogs not possessing pyridine nor sulfonamide analogs, **TM1_8** and **TM1_9**. To our knowledge, no chromone or dihydrobenzofuran analogs were reported as NAMPT inhibitors [13].

### 2.4. In Vitro NAMPT Activity and Cell Viability Assays

**Figure 6:**
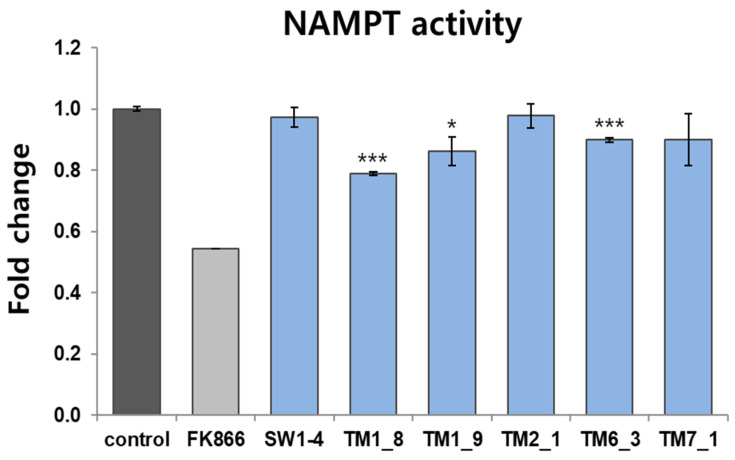
In vitro NAMPT activity assay. Compounds were incubated in the reaction mixture containing NAMPT and NAMPT activity was determined by measuring the absorbance at 450 nm as described in the “Materials and Methods” section. FK866 (100 nM) was used as a positive control. Data are expressed as mean ± SD. *, *p* < 0.05; ***, *p* < 0.001 versus vehicle-treated control (n = 3).

The selected compounds were tested for inhibitory activity using an in vitro NMAPT activity assay. The results showed that **TM1_8** significantly reduced NAMPT activity by approximately 0.80-fold (*p* < 0.005) (Figure 6). A milder but still significant inhibitory effect was observed for **TM6_3** (0.90-fold, *p* < 0.005) and **TM1_9** (0.86-fold, *p* < 0.05), whereas other compounds showed no significant inhibitory effect. FK866, a positive control, exhibited a strong inhibitory effect as expected.

**Figure 7:**
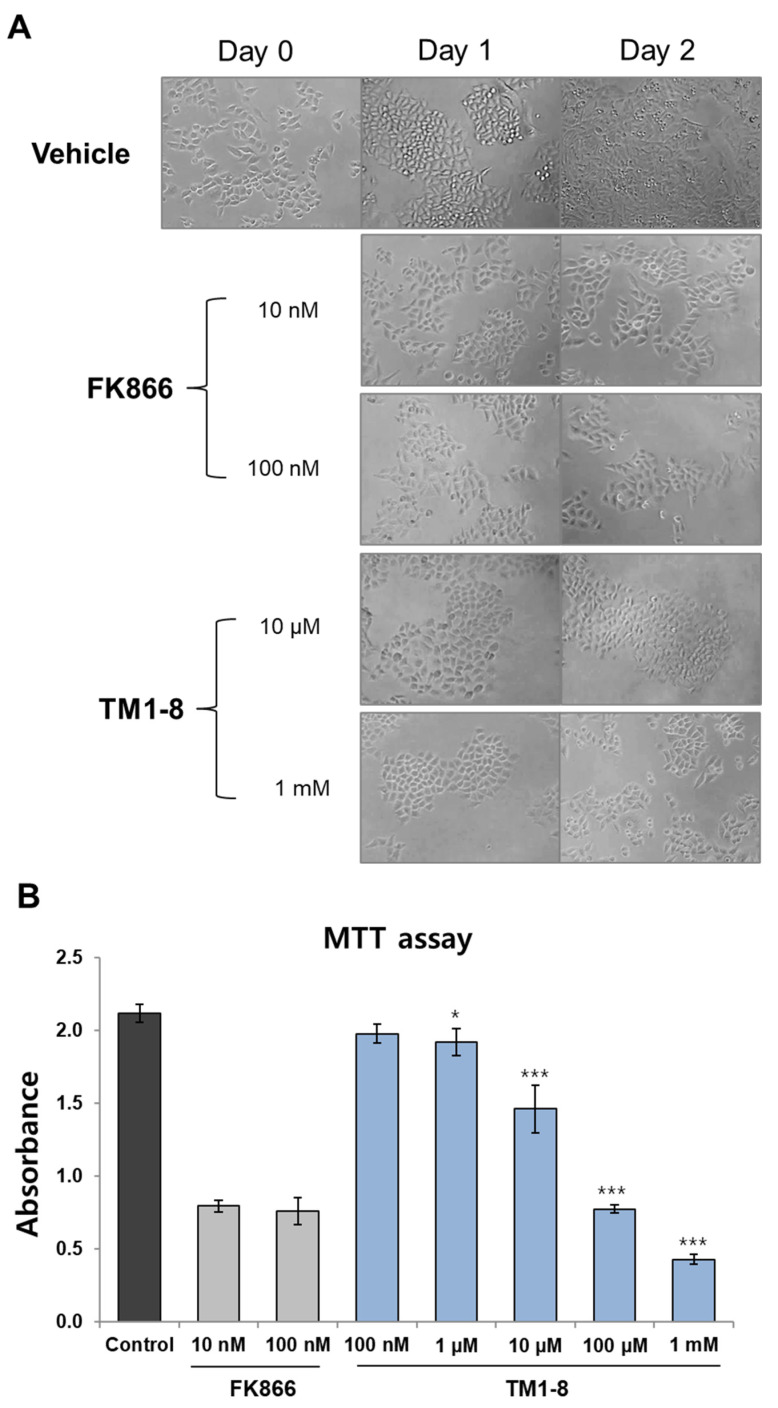
The effect of **TM1_8** on cell proliferation and viability. HeLa cells were cultured with or without **TM1_8**. (**A**) After incubation for 1 or 2 days, the effect of **TM1_8** on cell proliferation was observed. Photo magnification (×40). (**B**) An MTT assay was performed in cells treated with **TM1_8**. FK866 was used as a positive control. Data are expressed as mean ± SD. *, *p* < 0.05; ***, *p* < 0.001 versus vehicle-treated control (n = 3).

Based on these results, we further investigated the effect of **TM1_8** on cell proliferation. HeLa cells were seeded and grown for 2 days with or without **TM1_8** and cell growth was monitored daily. As shown in Figure 7A, vehicle-treated control cells proliferated normally; however, **TM1_8** strongly inhibited cell proliferation on days 1 and 2. This inhibitory effect was also observed in FK866-treated cells. To quantify the growth-inhibitory effect, we performed an MTT assay by incubating HeLa cells with various concentrations of **TM1_8**. We found that **TM1_8** downregulated cell viability in a concentration-dependent manner (Figure 7B), with an IC50 of 22.35 ± 1.26 μM (mean ± SE). As a positive control, FK866 also reduced cell viability; however, no further inhibition was observed at a higher concentration (100 nM), indicating that the maximum cytotoxic effect of FK866 was achieved at 10 nM. Although **TM1_8** exhibited weaker inhibitory activity than FK866 in cell-based assays, this is expected for an early-stage hit compound. Importantly, **TM1_8** represents a novel scaffold (chromone) distinct from conventional NAMPT inhibitors based on the pyridine or sulfonamide moiety, thereby expanding the chemical space of NAMPT-targeting ligands. Therefore, **TM1_8** would provide a structurally unique point for further optimization through structure–activity relationship studies.

**Figure 8:**
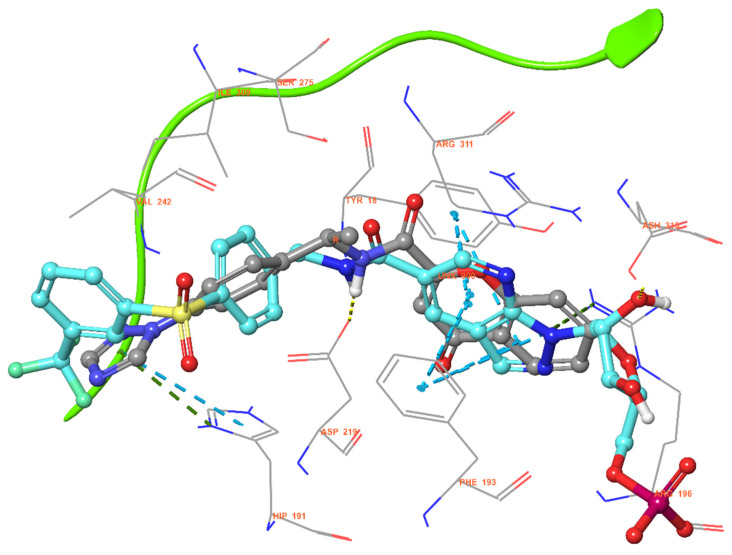
Docking mode of **TM1_8**. Superimposed structure of the docking mode of **TM1_8** and the co-crystal ligand (20T) of 4M6Q.

Further investigating the inhibitory activity of **TM1_8**, we performed a molecular docking analysis and compared its binding mode with the known potent inhibitor 20T (the original co-crystal ligand of 4M6Q). As shown in Figure 8, the chromone ring of **TM1_8** is positioned between ASP219 and TYR18, forming a stable pi–pi stacking interaction. The amide group (D6) in the linker region forms a key hydrogen bond with ASP219. Additionally, the triazole moiety participates in a pi–pi interaction with HIS191. Notably, **TM1_8** lacks the pi–cation interaction with ARG196 that is present in more potent derivatives like compound **1_14**. This absence provides a structural explanation for the relatively lower NAMPT inhibitory potency of **TM1_8** and offers critical insights for the further structural development and optimization of new inhibitors.

While conventional NAMPT inhibitors typically utilize a pyridine or nicotinamide moiety to mimic the substrate (NAM) at the catalytic center, **TM1_8** incorporates a triazole as a bioisostere. Furthermore, the chromane scaffold in **TM1_8** provides unique pi–pi stacking with surrounding aromatic residues. In terms of the linker, **TM1_8** features a shortened amide bond, which significantly differs from the longer, more flexible butyryl linker found in well-known inhibitors like FK866. These modifications collectively underscore the novelty of **TM1_8** as a distinct chemical entity in NAMPT inhibition.

## 3. Materials and Methods

### 3.1. Materials

All chemicals were purchased from ENAMINE (Middlesex County, NJ, USA) if there is no description and used for in vitro assay without further purification. NAMPT colorimetric assay kit (CY-1251) was purchased from Cyclex (Kyoto, Japan). FK866 was purchased from Cayman (Ann Arbor, MI, USA). 3-(4,5-dimethylthiazol-2-yl)-2,5-diphenyltetrazolium bromide (MTT) reagent was purchased from Sigma-Aldrich (St. Louis, MO, USA).

### 3.2. Dataset

Structural and biological information of NAMPT inhibitors was retrieved from ChEMBL [15,21,22]. Compounds were selected based on the following criteria: (1) identical assay conditions (identical assay ID), (2) removal of duplicate structures arising from salt forms or fragments, and (3) availability of exact IC_50_ values expressed in nM unit only, excluding data with ambiguous IC_50_s such as range-based values. As a result, 56 inhibitors were selected from ChEMBL as a dataset, and 29 inhibitors reported from two medicinal chemistry studies [16,23] were incorporated into the dataset (Table S1). To ensure adequate sampling of conformational flexibility, multiple 3D conformations were generated from SMILES of the inhibitors using OpenEye’s Omega 6.1.1.3 with default settings (Tables S2 and S3).

### 3.3. Docking

To obtain bioactive structures, the 3D conformers were docked into nine NAMPT structures (PDB IDs: 4KFO, 2GVJ, 4M6Q, 4LWW, 4LTS, 4N9B, 4N9C, 4N9D, 4N9E) using the Protein Preparation Wizard tool (Maestro, Version 10.5.014). NAMPT were selected from 33 available X-ray structures from the Protein Data Bank (PDB) [22,23,24,25].

All docking grids were made in the default conditions except for constraints of intermolecular hydrogen bonding between X-ray ligand and the apoprotein. The best docking scores of multi docking pose were selected based on GlideScore and Epik penalty [26].

Since docking conformers of the compounds in the dataset closely resembled X-ray conformers, we hypothesized a higher provability for X-ray conformers like bioactive conformers. Therefore, conformers with best docking score were produced in 4M6Q, 4LWW, 4LTS, 4N9B, 4N9D, and 4N9E. In case several conformers were presented in a single compound with a similar docking score and root mean square deviation (RMSD) of more than 2.0 Å, multi-conformers were used for generating pharmacophore models.

### 3.4. Building Pharmacophore Models

Pharmacophore modeling was conducted with the PHASE module (version 3.7, Schrödinger, New York, NY, USA) [27] under the default settings without additional structure-cleaning processes.

A total of 196 optimal conformers (from 85 compounds) were obtained based on docking and scoring results. To prepare the training and test sets, structure similarity maps were generated using Unity fingerprints in Sybyl (7.0) [28]. Compounds for the training set were selected based on the structural and activity diversity. The ratio between training and test sets was set to 7 to 3. The structures and biological activities of the NAMPT inhibitors used as training set and test set are shown in Table S1. pIC50, the negative logarithm of IC50, was calculated, and the activity threshold of 6.0 was used to distinguish active from inactive compounds. Finally, the training set included both the most and least active compounds.

Pharmacophore models were generated following four steps: (1) creation of pharmacophore sites from SMARTS patterns [29] of active compounds using the common features of hydrogen bond donor (D), hydrogen bond acceptor (A), hydrophobic region (H), negatively charged region (N), positively charged region (P) and aromatic rings (R); (2) identification of common pharmacophores using a tree-based partitioning algorithm (a binary decision tree with eight bins of 2 Å width); similar pharmacophores were grouped based on the distance between all pair of sites within each pharmacophore; (3) scoring and ranking of pharmacophore models generated from the partitioning procedures; and (4) construction of QSAR models.

To obtain the score, each pharmacophore model requires a reference ligand from the training set. The scoring was calculated by subtracting ‘the survival score of inactive compounds’ from ‘that of active compounds’ in the training set. The survival scoring function itself is defined in the following equation:

S = W_site_ S_site_ + W_vec_ S_vec_ + W_vol_ S_vol_ + W_sel_ S_sel_ + W m _rew_ − WEΔE + W_act_ A (1)

Every weighting coefficient (W) was set to ‘1.0’ as a default. A site score (S_site_) represents ‘root mean squared deviation (RMSD) between the reference (model) and compounds in the site-point positions’, a vector score (S_vec_) represents ‘the average cosine of the angles formed by corresponding pairs of vector features (acceptors, donors, and aromatic rings)’, and a volume score (S_vol_) represents ‘how much pairwise overlapped between the shape of a reference ligand and shape of compounds’. In order to further refine the ranking of compounds, a selectivity score (S_sel_) showing an empirical estimate of the rarity of a hypothesis, a reward (W_rew_; 1.0 by default) with m (the number of actives that match the hypothesis minus one), conformational energy (ΔE) of the reference ligand as a penalty for high-energy structures, and activity (A) of the reference ligand as a penalty for low activity are included.

### 3.5. Building QSAR Model from Optimal Pharmacophore Models

Based on the pharmacophore models, 3D-QSAR models were built and statistically evaluated. The conformers in a training set were first aligned to the set of pharmacophore features in the selected hypotheses using a standard least squares regression. The independent variables in the regression were set to ‘0’ or ‘1’ according to binary occupancies of the cubes (grid spacing 1.0 Å) by structural components of conformers and the activity values (pIC50) were used as the dependent variables. In order to reduce the number of the independent variables, a partial least squares (PLS) method was used with increasing number of PLS factors until detected as over-fitting. The PLS method involves finding a linear least squares relationship between the dependent variable and a special set of orthogonal factors that are linear combinations of independent variables. The optimal factor number was selected at the point where Q^2^ ceased to improve appreciably or began to decline despite continued improvement in R^2^, which is the canonical criterion for detecting overfitting in PLS-based QSAR. The pharmacophore feature-based QSAR model permitted duplicate feature definition of atoms in conformers. The independent variables could also be filtered using a t-value filter to eliminate independent variables whose regression coefficients are overly sensitive to small changes in the training set composition (cutoff of t-value: 2.0).

### 3.6. Validation of QSAR Models

In general, validation is carried out to check the robustness of a model with predictive power. Representative statistical measurements include coefficient of determination (r^2^), leave-n-out (LNO), cross-validation coefficient (q^2^ or r_cv_^2^), least squares error (LSE), and lack of fit measure (LOF). In 3D-QSAR models, a model is accepted if the model can satisfy all of the following conditions [29]:

q^2^ > 0.5, r^2^ > 0.6, [(r^2^ − r_0_^2^)/r^2^] < 0.1, [(r^2^ − r′_0_^2^)/r^2^] < 0.1, 0.85 ≤ k ≤ 1.15 and r_m_^2^ > 0.5

The coefficient of determination (r_0_^2^, square of Pearson r in zero-order regression) value from training set is calculated by the following formula against dependent variable (*Y*):

r_0_^2^ =1 − ∑(*Y*_predicted_ − *Y*_observed_)^2^/∑(*Y*_observed_ − *Y*_mean_)^2^ (2)

The q^2^ value is calculated by the same formula with (2) except for data. In our study, the q^2^ value is calculated from the test set as an external validation. The Pearson correlation coefficient, r, is calculated by the following formula:

r = ∑(*Y*_predicted_ − *Ȳ*_predicted_) (*Y*_observed_ − *Ȳ*_observed_)/√∑(*Y*_predicted_ − *Ȳ*_predicted_)^2^ √∑(*Y*_observed_ − *Ȳ*_observed_)^2^ = Covariance(*Y*_predicted_, *Y*_observed_)/SD(*Y*_predicted_) SD(*Y*_observed_) (3)

Slope k from the regression of *Y*_observed_ against *Y*_predicted_ through the origin (Y^RO^ =k *Y*_predicted_) is calculated as follows:

k = ∑(*Y*_predicted_ × *Y*_observed_)/∑*Y*_predicted_ (4)

Another statistical parameter r_m_^2^ was defined as follows:

r_m_^2^ = r^2^(1 − √|r^2^ − r_0_^2^|) (5)

For validation of our models, internal cross validation directly was not used. Merely, leave-one-out (LOO) technique was used for assessing the stability of a model to changes in the training set. The stability of a model was defined as the r^2^ value and computed between the LOO predictions and the predictions from the model built on the full training set (maximum value = 1). In addition, the F-value was used as the ratio of the model variance to the observed activity variance. In a model variance, degrees of freedom were PLS factors (m) and in the observed activity variance, it was ‘ligand number (n) − m − 1’. From the F-value and significant value, the *p*-value was determined.

### 3.7. Pharmacophore Model-Based Virtual Screening

The validated pharmacophore-based QSAR models were used to screen the ZINC database. One conformer of compounds in the ZINC database was amplified to multi conformers under the option of (1) within 10 conformers per one rotatable bond in one compound, (2) within the total of 100 conformers per one compound, (3) permission of amide bond variance, and (4) energy window range of 10 Kcal/mol. In matching results with pharmacophore models, intersite distance matching tolerance with pharmacophore features was 2.0 Å, the number of matching features in a hit was two, and the number of hit compounds was limited to ‘1000’. Every hit possessed a fitness score, as shown in Equation 1, and predicted activity.

### 3.8. In Vitro NAMPT Activity Assay

NAMPT activity was determined using the NAMPT Colorimetric Assay Kit according to the manufacturer’s instructions (cat. CY-12511, CycLex, Woburn, MA, USA). Briefly, NAMPT catalyzed the conversion of nicotinamide to nicotinamide mononucleotide (NMN), which was sequentially converted to NAD by mononucleotide adenylyltransferase 1 (NMNAT). In the second reaction, the resulting NAD was reduced to NADH by alcohol dehydrogenase (ADH), and NADH led to the formation of WST-1 via diaphorase. After 30 min of incubation, the absorbance of WST-1-formazan was measured at 450 nm. Each compound was added to the reaction at a final concentration of 100 μM and 10 nM FK866 was used as a positive control.

### 3.9. Cell Culture and Viability Assay

HeLa cells were maintained in Dulbecco’s modified Eagle’s medium (DMEM) supplemented with 10% fetal bovine serum (Invitrogen, Carlsbad, CA, USA). The cells were grown in a humidified incubator at 37 °C with 5% CO_2_. Cell morphology was observed using a microscope (Eclipse TS100-F, Nikon, Tokyo, Japan). For the MTT assay, 1 × 10^5^ cells were seeded, and **TM1_8** or FK866 was added the following day. After 3 days, the cells were incubated with MTT solution at 37 °C for 4 h. The absorbance was measured at 590 nm using a microplate reader (Synergy H1 hybrid reader, BioTek Instruments, Winooski, VT, USA).

## 4. Conclusions

NAMPT plays a key role as a rate-limiting enzyme in regulating cellular NAD+ level. In this study, we employed structure-based virtual screening and discovered novel effective inhibitor candidates against NAMPT. Pharmacophore models were generated by analyzing a large number of previously validated ligand structures and multiple candidate models were selected with the criteria of R^2^ > 0.7 and stability values over 0.9. Among candidate models, ADDRRR.146 was chosen as the best based on a well-known validation process, from which eight compounds were derived as probable active analogs.

The NAMPT inhibitory activities of these candidates were further validated by in vitro enzymatic assays and by cell viability assays. Although the effect of **TM1_8** was not as strong as well-known potent NAMPT inhibitor FK866, **TM1_8** reduced NAMPT activity and cell viability. It is noticeable that **TM1_8** represents a novel active scaffold which has not been reported ever in NAMPT inhibitors. Therefore, it is concluded that our 3D-QSAR-based approach using big data provides the efficient way to generate novel structures for drug discovery. Our new method introduced here has a great advantage and merit from the viewpoint of time and cost. Additionally, we suggest that this approach might be more plausible and effective in cases where the structures in the training set are highly flexible or structurally diverse.

## Figures and Tables

**Table 1:** Various statistic pharmacophore model results.

ID	SD	R^2^	F	P	Stability	RMSE	Q^2^	Pearson
ADDRRR.146	0.6941	0.8514	171.8	5.94 × 10^−14^	0.9595	0.8093	0.6789	0.8579
AADRRR.1198	0.7001	0.8488	168.4	7.69 × 10^−14^	0.9841	0.8330	0.6599	0.8592
AADRRR.718	0.7001	0.8488	168.4	7.69 × 10^−14^	0.9841	0.8330	0.6599	0.8592
AADDRR.134	0.7337	0.8294	150.7	1.94 × 10^−13^	0.989	0.8472	0.6482	0.8223
AADDRR.312	0.7575	0.7693	90.0	4.32 × 10^−10^	0.9136	0.8767	0.6326	0.8037
AADDRR.164	0.7575	0.7693	90.0	4.32 × 10^−10^	0.9136	0.8767	0.6326	0.8037
AADRRR.719	0.7273	0.8368	153.9	2.43 × 10^−13^	0.9870	0.8732	0.6263	0.8236
AADRRR.1199	0.7273	0.8368	153.9	2.43 × 10^−13^	0.9870	0.8732	0.6263	0.8236
ADDRRR.83	0.7039	0.8471	166.3	9.08 × 10^−14^	0.9818	0.8806	0.6199	0.8266
AADRRR.1161	0.9597	0.7081	75.2	8.59 × 10^−10^	0.9735	0.8835	0.6174	0.7982
AADRRR.717	0.6702	0.8614	186.5	2.06 × 10^−14^	0.9807	0.8852	0.6159	0.8415

SD: standard deviation; R^2^: the coefficient of determination; F: the ratio of model variance to the observed activity variance; the larger F, the more statistically significant in regression; P: significance level of variance ratio; smaller values represent a greater degree of confidence; Stability: the stability of the model predictions with changes in the training set composition [14]; RMSE: Root Mean-Square Error, similar to SD but typically used for the test set to evaluate external validation; Q^2^: predicted squared correlated coefficient.

## Data Availability

The original contributions presented in this study are included in the article/Supplementary Material. Further inquiries can be directed to the corresponding authors.

## Supplementary Materials

The following supporting information can be downloaded at: https://www.mdpi.com/article/10.3390/molecules31101773/s1, Table S1: Structures and biological activities of the NAMPT inhibitors used in this study. Table S2. Generated phase pharmacophore models from multi-conformers of NAMPT inhibitors. Table S3. Generated phase pharmacophore models from one conformer of NAMPT inhibitors. Table S4. Prediction result of data set through the model (ADDRRR146). Figure S1: Validation of the docking protocol via redocking.

## References

[1] Chiarugi A., Dolle C., Felici R., Ziegler M. (2012). The NAD metabolome—A key determinant of cancer cell biology. Nat. Rev. Cancer.

[2] Heske C.M. (2019). Beyond Energy Metabolism: Exploiting the Additional Roles of NAMPT for Cancer Therapy. Front. Oncol..

[3] Tan B., Young D.A., Lu Z.H., Wang T., Meier T.I., Shepard R.L., Roth K., Zhai Y., Huss K., Kuo M.S. (2013). Pharmacological inhibition of nicotinamide phosphoribosyltransferase (NAMPT), an enzyme essential for NAD+ biosynthesis, in human cancer cells: Metabolic basis and potential clinical implications. J. Biol. Chem..

[4] Imai S. (2009). Nicotinamide phosphoribosyltransferase (Nampt): A link between NAD biology, metabolism, and diseases. Curr. Pharm. Des..

[5] Li X.Q., Lei J., Mao L.H., Wang Q.L., Xu F., Ran T., Zhou Z.H., He S. (2019). NAMPT and NAPRT, Key Enzymes in NAD Salvage Synthesis Pathway, Are of Negative Prognostic Value in Colorectal Cancer. Front. Oncol..

[6] Lucena-Cacace A., Otero-Albiol D., Jimenez-Garcia M.P., Munoz-Galvan S., Carnero A. (2018). NAMPT Is a Potent Oncogene in Colon Cancer Progression that Modulates Cancer Stem Cell Properties and Resistance to Therapy through Sirt1 and PARP. Clin. Cancer Res..

[7] Zhang H., Zhang N., Liu Y., Su P., Liang Y., Li Y., Wang X., Chen T., Song X., Sang Y. (2019). Epigenetic Regulation of NAMPT by NAMPT-AS Drives Metastatic Progression in Triple-Negative Breast Cancer. Cancer Res..

[8] Meram A.T., Alzubaidi Y., Cotelingam J., Ghali G., Lopez L., Coppola D., Shackelford R. (2019). Nicotinamide Phosphoribosyl Transferase Is Increased in Osteosarcomas and Chondrosarcomas Compared to Benign Bone and Cartilage. Anticancer Res..

[9] (2022). ClinicalTrials.gov [Internet]. https://clinicaltrials.gov/study/NCT00724841.

[10] Galli U., Colombo G., Travelli C., Tron G.C., Genazzani A.A., Grolla A.A. (2020). Recent Advances in NAMPT Inhibitors: A Novel Immunotherapic Strategy. Front. Pharmacol..

[11] Galli U., Travelli C., Massarotti A., Fakhfouri G., Rahimian R., Tron G.C., Genazzani A.A. (2013). Medicinal chemistry of nicotinamide phosphoribosyltransferase (NAMPT) inhibitors. J. Med. Chem..

[12] Xu T.Y., Zhang S.L., Dong G.Q., Liu X.Z., Wang X., Lv X.Q., Qian Q.J., Zhang R.Y., Sheng C.Q., Miao C.Y. (2015). Discovery and characterization of novel small-molecule inhibitors targeting nicotinamide phosphoribosyltransferase. Sci. Rep..

[13] Wei Y., Xiang H., Zhang W. (2022). Review of various NAMPT inhibitors for the treatment of cancer. Front. Pharmacol..

[14] Zhou N., Xu Y., Liu X., Wang Y., Peng J., Luo X., Zheng M., Chen K., Jiang H. (2015). Combinatorial Pharmacophore-Based 3D-QSAR Analysis and Virtual Screening of FGFR1 Inhibitors. Int. J. Mol. Sci..

[15] Zheng X., Bair K.W., Bauer P., Baumeister T., Bowman K.K., Buckmelter A.J., Caligiuri M., Clodfelter K.H., Feng Y., Han B. (2013). Identification of amides derived from 1H-pyrazolo[3,4-b]pyridine-5-carboxylic acid as potent inhibitors of human nicotinamide phosphoribosyltransferase (NAMPT). Bioorg. Med. Chem. Lett..

[16] Dragovich P.S., Zhao G., Baumeister T., Bravo B., Giannetti A.M., Ho Y.C., Hua R., Li G., Liang X., Ma X. (2014). Fragment-based design of 3-aminopyridine-derived amides as potent inhibitors of human nicotinamide phosphoribosyltransferase (NAMPT). Bioorg. Med. Chem. Lett..

[17] Shin W.-H., Zhu X., Bures M.G., Kihara D. (2015). Three-dimensional compound comparison methods and their application in drug discovery. Molecules.

[18] Hawkins P.C., Skillman A.G., Nicholls A. (2007). Comparison of shape-matching and docking as virtual screening tools. J. Med. Chem..

[19] Kim H., Jang C., Yadav D.K., Kim M.-h. (2017). The comparison of automated clustering algorithms for resampling representative conformer ensembles with RMSD matrix. J. Cheminform..

[20] Maia E.H.B., Assis L.C., de Oliveira T.A., da Silva A.M., Taranto A.G. (2020). Structure-Based Virtual Screening: From Classical to Artificial Intelligence. Front. Chem..

[21] You H., Youn H.S., Im I., Bae M.H., Lee S.K., Ko H., Eom S.H., Kim Y.C. (2011). Design, synthesis and X-ray crystallographic study of NAmPRTase inhibitors as anti-cancer agents. Eur. J. Med. Chem..

[22] Lockman J.W., Murphy B.R., Zigar D.F., Judd W.R., Slattum P.M., Gao Z.H., Ostanin K., Green J., McKinnon R., Terry-Lorenzo R.T. (2010). Analogues of 4-[(7-Bromo-2-methyl-4-oxo-3H-quinazolin-6-yl)methylprop-2-ynylamino]-N-(3-pyridy lmethyl)benzamide (CB-30865) as potent inhibitors of nicotinamide phosphoribosyltransferase (Nampt). J. Med. Chem..

[23] Zheng X., Baumeister T., Buckmelter A.J., Caligiuri M., Clodfelter K.H., Han B., Ho Y.C., Kley N., Lin J., Reynolds D.J. (2014). Discovery of potent and efficacious cyanoguanidine-containing nicotinamide phosphoribosyltransferase (Nampt) inhibitors. Bioorg. Med. Chem. Lett..

[24] Khan J.A., Tao X., Tong L. (2006). Molecular basis for the inhibition of human NMPRTase, a novel target for anticancer agents. Nat. Struct. Mol. Biol..

[25] Zheng X., Bauer P., Baumeister T., Buckmelter A.J., Caligiuri M., Clodfelter K.H., Han B., Ho Y.C., Kley N., Lin J. (2013). Structure-based identification of ureas as novel nicotinamide phosphoribosyltransferase (Nampt) inhibitors. J. Med. Chem..

[26] Friesner R.A., Banks J.L., Murphy R.B., Halgren T.A., Klicic J.J., Mainz D.T., Repasky M.P., Knoll E.H., Shelley M., Perry J.K. (2004). Glide: A new approach for rapid, accurate docking and scoring. 1. Method and assessment of docking accuracy. J. Med. Chem..

[27] Dixon S.L., Smondyrev A.M., Knoll E.H., Rao S.N., Shaw D.E., Friesner R.A. (2006). PHASE: A new engine for pharmacophore perception, 3D QSAR model development, and 3D database screening: 1. Methodology and preliminary results. J. Comput. Aided-Mol. Des..

[28] Cereto-Massagué A., Ojeda M.J., Valls C., Mulero M., Garcia-Vallvé S., Pujadas G. (2015). Molecular fingerprint similarity search in virtual screening. Methods.

[29] Golbraikh A., Tropsha A. (2002). Beware of q2!. J. Mol. Graph. Model..

