# Supplementary material for: Pharmacophore-Based QSAR Model of Multi Scaffolds as NAMPT Inhibitors & Scaffold Diversity Analysis
Source: Molecules. 2026 May 21;31(10):1773. doi: 10.3390/molecules31101773 (PMC13209195; doi:10.3390/molecules31101773)
Supplement: Supplementary file 1 [file molecules-31-01773-s001.zip › molecules-4282243-supplementary.pdf]

Pharmacophore-based QSAR model of multi scaffolds as NAMPT inhibitors & scaffold diversity analysis

Sujin Lee [1,*], Mei Zheng [2], Kang Kim [2] and Kwang-Hoon Chun [2,*]

[1] College of Pharmacy, Daegu Catholic University, Gyeongsan 38430, Republic of Korea
[2] Gachon Institute of Pharmaceutical Sciences, College of Pharmacy, Gachon University, Incheon 21936, Republic of Korea
* Correspondence: sujinlee20@cu.ac.kr (S.L.); khchun@gachon.ac.kr (K.-H.C.)

Table S1. Structures and biological activities of the NAMPT inhibitors used in this study.

The dataset of the training set and test set (*) compounds

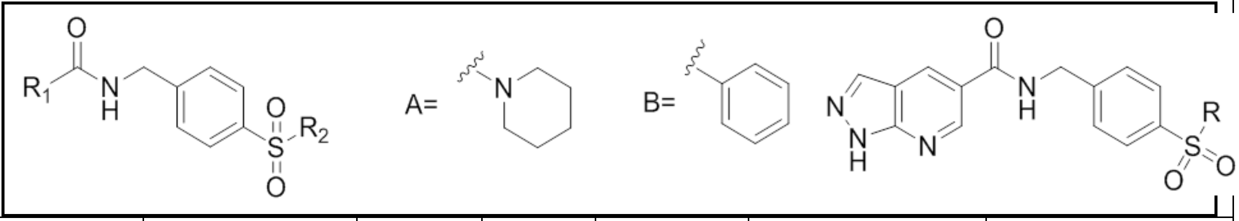

Molecule ID	R_1	R_2	IC50	pIC50	pIC50 (ADDRRR.146)	pIC50 (AADRRR.133)
1_03*	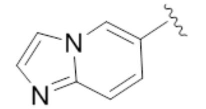	A	0.028	7.552842	7.144595	7.128349
1_09*	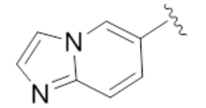	B	0.018	7.744727	7.176042	7.209952
1_11*	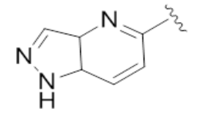	B	1.3	5.886057	6.990747	7.09919
1_13*	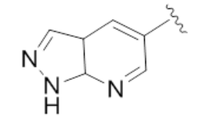	A	0.1	7	6.924507	7.11311
1_14*	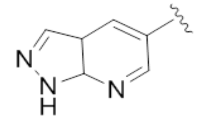	B	0.054	7.267606	6.924507	7.325168
1_15*	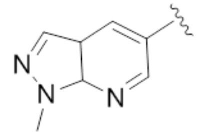	B	1.7	5.769551	6.87092	7.068059
1_16*	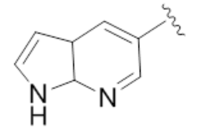	B	1.9	5.721246	6.584759	7.243565
1_17	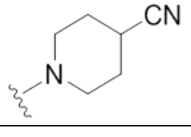		0.41	6.387216	6.924507	7.11311
1_18	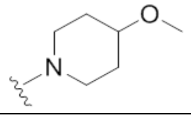		0.4	6.39794	6.856021	7.229173

1_19	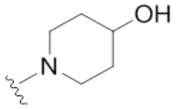	0.41	6.387216	6.924507	7.243565
1_20	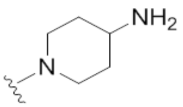	0.39	6.408935	6.924507	7.11311
1_21	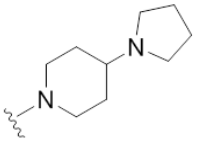	0.3	6.522879	6.818994	7.31373
1_22*	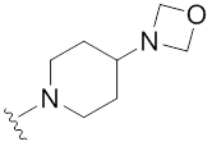	0.44	6.356547	6.924507	7.11311
1_23	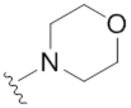	0.37	6.431798	6.88748	7.11311
1_24	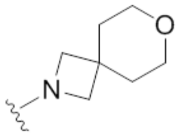	0.11	6.958607	6.771745	6.997894
1_25	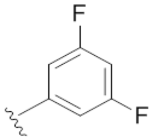	0.0079	8.102373	6.924507	7.194713
1_26	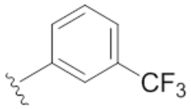	0.0061	8.21467	6.88748	7.194713

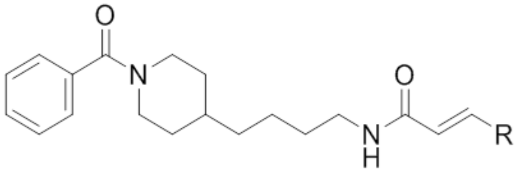

Molecule ID	R	IC50	pIC50	pIC50 (ADDRRR.146)	pIC50 (AADRRR.133)
FK866	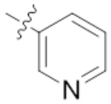	0.68		-	7.110925

2_07*	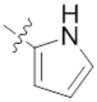	1.29	5.88941	6.706297	7.251897

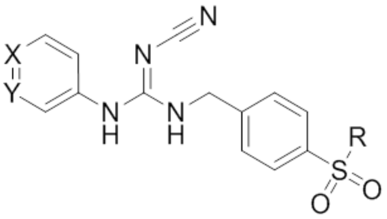

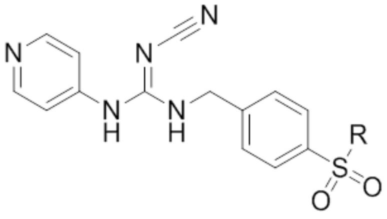

Molecule ID	X Y	R	IC50	pIC50	pIC50 (ADDRRR.146)	pIC50 (AADRRR.133)
4_04*	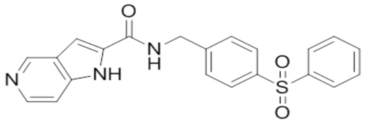		0.029	8.60206<br>7.537602	6.318416<br>6.91226	6.829728
4_05*	N CH	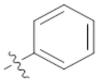	0.0025	8.619789<br>8.60206	8.828158<br>8.127835	8.264518<br>7.84399
4_06	N CH	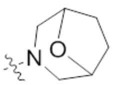	0.0024	8.657577<br>8.619789	8.73069<br>8.292607	8.134778<br>7.978173
4_07*	CH N	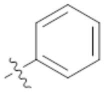	0.0022	8.657577<br>8.356547	8.63843<br>8.828158	8.047927<br>7.762743
4_08*	CH N	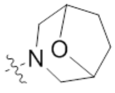	0.0044	8.124939<br>8.356547	8.832197<br>8.628035	8.237991
4_09*	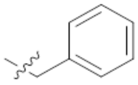		0.0075	8.267606<br>8.124939	8.841609<br>8.36135	7.865029<br>7.886839
4_10	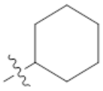		0.0054	8.69897<br>8.267606	8.696378<br>8.055559	7.916223<br>7.864189
4_11	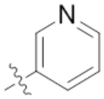		0.002	8.769551<br>8.69897	8.608236<br>8.578781	7.886839<br>7.882025
4_12	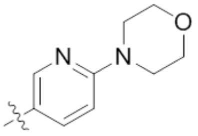		0.0017	8.619789<br>8.769551	8.803142<br>7.998053	7.846653<br>8.071137

4_13*	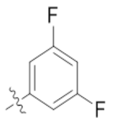	0.0006	8.356547<br>9.221849	8.950612<br>8.03879	8.249766<br>7.84399
4_14*	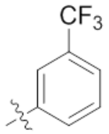	0.0024	8.721246<br>8.619789	8.738058<br>7.705485	7.846653<br>6.957107
4_15	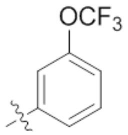	0.0044	8.443697	8.697433	8.264518
4_16	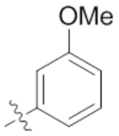	0.0019	7.537602<br>8.721246	8.738058<br>7.895558	7.886839<br>5.712667
4_17*	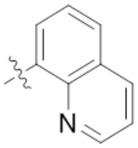	0.0036	9.221849<br>8.443697	8.744976<br>8.378781	8.134778<br>7.734475

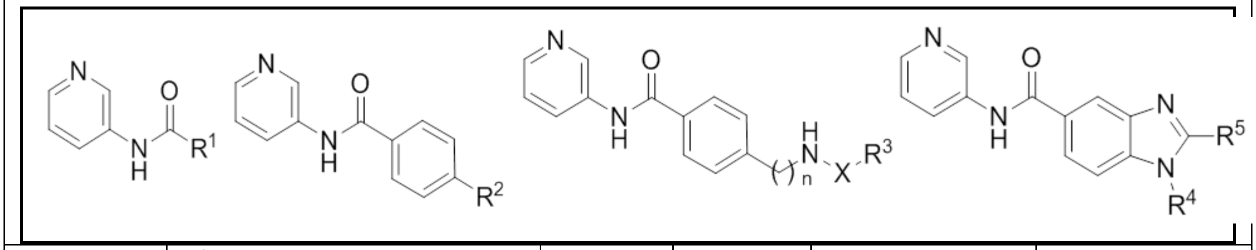

Molecule ID	R[1]	IC50	pIC50	pIC50 (ADDRRR.146)	pIC50 (AADRRR.133)
5_07*	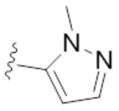	91	4.040959	4.160529	4.063013
5_10*	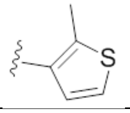	34	4.468521	4.119721	4.089653
5_12*	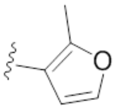	38	4.420216	4.119721	4.011991
5_14*	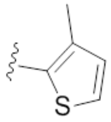	50	4.30103	4.313892	4.617362

5_52	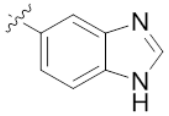	1.5	5.823909	6.343656	5.13145
5_53	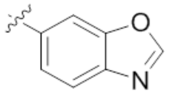	9.1	5.040959	4.971996	5.189947
5_54	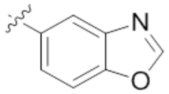	3.4	5.468521	4.971996	5.175555
5_55*	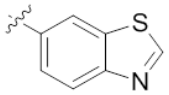	26	4.585027	4.989699	5.10587
5_56	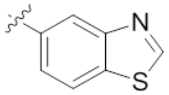	4.1	5.387216	5.587285	5.526421
5_57	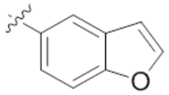	21	4.677781	4.971996	5.202284
5_58	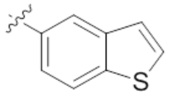	21	4.677781	5.191974	5.513608

Molecule ID	R^2	IC50	pIC50	pIC50 (ADDRRR.146)	pIC50 (AADRRR.133)
5_25	H	13	4.886057	4.315708	4.652186
5_26	CH_3	13	4.886057	4.262372	4.919104
5_27	Ph	1	6	4.356516	4.928776
5_28	Cyclohexyl	1	6	4.356516	4.95641
5_30	NHBoc	0.42	6.376751	5.519407	5.230307
5_31	$NHSO_2CH_3$	10.3	4.987163	4.880881	4.941974
5_33	CH_2NHBoc	13	4.886057	4.716774	5.228472
5_34	CH_2NHCbz	3.3	5.481486	4.934438	8.164906
5_35	$CH_2NHSO_2CH_3$	5	5.30103	4.778155	4.548178

Molecule ID	R^3	X	n	IC50	pIC50	pIC50 (ADDRRR.146)	pIC50 (AADRRR.133)
5_36*	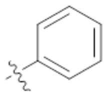	C=O	0	0.61	6.21467	5.543082	4.773823
5_37		C=O	1	25	4.60206	5.255393	5.532788
5_39		SO_2	1	0.21	6.677781	6.120437	6.15391
5_40	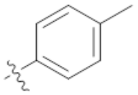	C=O	0	1.5	5.823909	5.15224	5.604863
5_41		C=O	1	40	4.39794	4.997052	4.900729
5_42*		SO_2	0	83	4.080922	4.952339	4.879658

5_43*		SO_2 1	0.014	7.853872	6.173773	6.15391
5_44	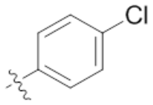	C=O 0	0.64	6.19382	5.152141	5.580727
5_45		C=O 1	3.1	5.508638	5.03354	5.030266
5_46		SO_2 0	62	4.207608	4.952339	4.738049
5_47		SO_2 1	0.019	7.721246	4.823274	4.798862
5_48	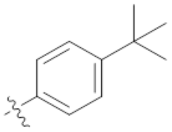	C=O 0	0.29	6.537602	5.328384	5.521559
5_49*		C=O 1	80	4.09691	4.973438	5.595629
5_50		SO_2 0	36	4.443697	4.75551	4.848511
5_51		SO_2 1	0.019	7.721246	6.485688	5.687867

Molecule ID	R^4	R^5	IC50	pIC50	pIC50 (ADDRRR.146)	pIC50 (AADRRR.133)
5_61	CH_3	H	1.4	5.853872	4.971996	5.325547
5_62	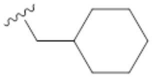	H	3.7	5.431798	5.043035	5.099145
5_63	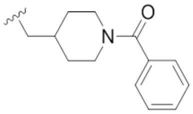	H	0.015	7.823909	7.22003	5.684961
5_64	CH_3	CH_3	6.1	5.21467	5.052148	5.086808
5_65*	CH_3	Ph	14	4.853872	5.38707	5.655442

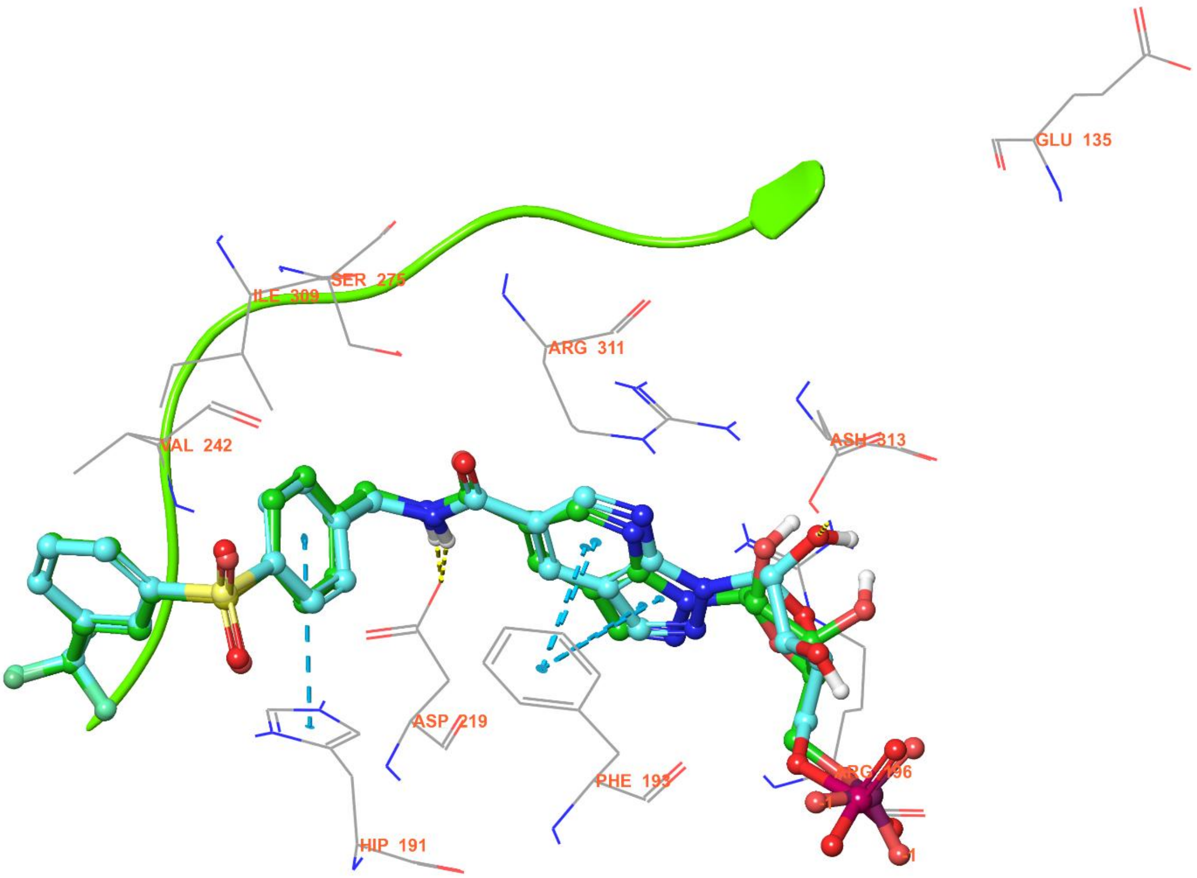

Figure S 1. Validation of the docking protocol via redocking. The co-crystallized ligand (20T, shown in green) was redocked into the generated pharmacophore model to verify the binding mode. The predicted pose (sky blue) demonstrated high structural overlap with the original crystal conformation, confirming the reliability of the docking parameters used in this study.

Table S2: Generated phase pharmacophore models from multi-conformers of NAMPT inhibitors

ID	# Factors	SD	R^2	F	P	Stability	RMSE	Q^2	Pearson-R
**ADDRRR.146**	1	0.6941	0.8514	171.8	5.94E-14	0.9595	0.8093	0.6789	0.8579
**AADRRR.1198**	1	0.7001	0.8488	168.4	7.69E-14	0.9841	0.833	0.6599	0.8592
**AADRRR.718**	1	0.7001	0.8488	168.4	7.69E-14	0.9841	0.833	0.6599	0.8592
**AADDRR.134**	1	0.7337	0.8294	150.7	1.94E-13	0.989	0.8472	0.6482	0.8223
**AADDRR.312**	1	0.7575	0.7693	90	4.32E-10	0.9136	0.8767	0.6326	0.8037
**AADDRR.164**	1	0.7575	0.7693	90	4.32E-10	0.9136	0.8767	0.6326	0.8037
**AADRRR.719**	1	0.7273	0.8368	153.9	2.43E-13	0.987	0.8732	0.6263	0.8236
**AADRRR.1199**	1	0.7273	0.8368	153.9	2.43E-13	0.987	0.8732	0.6263	0.8236
**ADDRRR.83**	1	0.7039	0.8471	166.3	9.08E-14	0.9818	0.8806	0.6199	0.8266
**AADRRR.1161**	1	0.9597	0.7081	75.2	8.59E-10	0.9735	0.8835	0.6174	0.7982
**AADRRR.717**	1	0.6702	0.8614	186.5	2.06E-14	0.9807	0.8852	0.6159	0.8415
**AADRRR.1197**	1	0.6702	0.8614	186.5	2.06E-14	0.9807	0.8852	0.6159	0.8415
**AADDRR.257**	1	0.8275	0.783	111.8	8.30E-12	0.9905	0.8873	0.6141	0.8015
**AAADRR.228**	1	0.868	0.7612	98.8	3.70E-11	0.9828	0.8907	0.6111	0.8004
**AAADRR.588**	1	0.868	0.7612	98.8	3.70E-11	0.9828	0.8907	0.6111	0.8004
**ADDRRR.10**	1	0.7813	0.8065	129.2	1.38E-12	0.9804	0.8971	0.6055	0.8254
**AADRRR.1160**	1	0.887	0.7506	93.3	7.29E-11	0.9814	0.8979	0.6048	0.7933
**AADDRR.309**	1	0.7724	0.8109	132.9	9.63E-13	0.9832	0.9047	0.5988	0.8067
**AADDRR.161**	1	0.7724	0.8109	132.9	9.63E-13	0.9832	0.9047	0.5988	0.8067
**AADRRR.1155**	1	0.8852	0.7516	93.8	6.83E-11	0.9769	0.9208	0.5844	0.7796
**ADRRRR.231**	1	0.8193	0.7929	114.9	8.89E-12	0.9506	0.9228	0.5826	0.8183
**AADRRR.1156**	1	0.9525	0.7124	76.8	6.79E-10	0.9758	0.9291	0.5768	0.7721
**AAADRR.554**	1	1.0328	0.6619	60.7	8.64E-09	0.9848	0.9378	0.5689	0.755
**AAADRR.204**	1	1.0328	0.6619	60.7	8.64E-09	0.9848	0.9378	0.5689	0.755
**ADRRRR.369**	1	0.986	0.7001	70	2.43E-09	0.9406	0.9493	0.5583	0.7688
**AAAADR.83**	1	0.89	0.7557	92.8	1.09E-10	0.9419	0.9509	0.5568	0.7619

**AAAADR.81**	1	0.89	0.7557	92.8	1.09E-10	0.9419	0.9509	0.5568	0.7619
**AAADRR.206**	1	0.9198	0.7318	84.6	2.27E-10	0.9882	0.954	0.5538	0.7519
**AAADRR.556**	1	0.9198	0.7318	84.6	2.27E-10	0.9882	0.954	0.5538	0.7519
**ADDRRR.7**	1	0.7587	0.8176	138.9	5.51E-13	0.9701	0.9667	0.5419	0.8181
**AAAADR.79**	1	0.8508	0.7767	104.3	2.78E-11	0.9408	0.9763	0.5328	0.7512
**AAAADR.80**	1	0.8508	0.7767	104.3	2.78E-11	0.9408	0.9763	0.5328	0.7512
**ADDRRR.145**	1	0.723	0.8343	156.1	1.23E-13	0.9835	0.9875	0.522	0.7914
**ADDRRR.81**	1	0.8091	0.7925	118.4	4.10E-12	0.9778	0.9904	0.5192	0.7862
**ADDRRR.6**	1	0.8378	0.7775	108.4	1.22E-11	0.9692	0.9915	0.5181	0.798
**ADRRRR.32**	1	0.9473	0.7156	78	5.72E-10	0.9663	0.9989	0.5109	0.7557
**ADRRRR.230**	1	0.8995	0.7504	90.2	1.50E-10	0.9707	1.0056	0.5044	0.7541
**AADRRR.235**	1	0.8488	0.7716	104.7	1.84E-11	0.9778	1.0062	0.5038	0.7568
**ADRRRR.371**	1	0.8271	0.7889	112.1	1.19E-11	0.9456	1.0101	0.4999	0.7705
**AADDRR.322**	1	0.8655	0.7046	62	2.37E-08	0.8541	1.0166	0.497	0.7241
**ADDRRR.82**	1	0.874	0.7579	97	4.59E-11	0.9839	1.0141	0.4959	0.7458
**AAADDR.84**	1	0.9507	0.7135	77.2	6.40E-10	0.9771	1.0155	0.4945	0.7348
**AAADDR.167**	1	0.9507	0.7135	77.2	6.40E-10	0.9771	1.0155	0.4945	0.7348
**ADRRRR.233**	1	0.8955	0.7526	91.3	1.31E-10	0.9704	1.0226	0.4874	0.754
**ADRRRR.234**	1	0.9123	0.7433	86.9	2.30E-10	0.9689	1.0243	0.4858	0.722
**AADRRR.236**	1	0.8316	0.7808	110.4	9.67E-12	0.9748	1.0313	0.4787	0.7487
**AADRRR.234**	1	0.838	0.7774	108.3	1.23E-11	0.977	1.0313	0.4786	0.7466
**AAADDR.94**	1	0.7854	0.8045	127.6	1.62E-12	0.9603	1.0398	0.4701	0.7615
**AAADDR.177**	1	0.7854	0.8045	127.6	1.62E-12	0.9603	1.0398	0.4701	0.7615
**ADRRRR.370**	1	1.0103	0.6852	65.3	5.10E-09	0.9614	1.043	0.4668	0.7077
**AADDRR.148**	1	0.9634	0.7058	74.4	9.69E-10	0.9709	1.0497	0.4599	0.6955
**ADRRRR.31**	1	0.8156	0.7891	116	5.28E-12	0.9745	1.0539	0.4556	0.7511
**AAADDR.89**	1	1.0568	0.646	56.6	1.78E-08	0.9862	1.0577	0.4516	0.6882
**AAADDR.172**	1	1.0568	0.646	56.6	1.78E-08	0.9862	1.0577	0.4516	0.6882

**AAADRR.1039**	1	0.9332	0.6566	49.7	1.73E-07	0.865	1.0886	0.4336	0.6854
**AAADRR.1032**	1	1.0177	0.6241	46.5	2.09E-07	0.8671	1.0921	0.4299	0.6787
**ADDRRR.84**	1	0.8208	0.7922	114.4	9.38E-12	0.9714	1.0801	0.4281	0.7703
**AADDRR.286**	1	0.9514	0.7131	77.1	6.54E-10	0.9683	1.0878	0.42	0.6874
**ADDRRR.147**	1	0.6466	0.871	202.6	7.01E-15	0.9788	1.1021	0.4047	0.7707
**AADDRR.264**	1	0.9074	0.739	87.8	1.48E-10	0.9616	1.114	0.3917	0.7074
**AAADDR.169**	1	0.8223	0.7857	113.6	6.82E-12	0.9836	1.1151	0.3905	0.7123
**AAADDR.86**	1	0.8223	0.7857	113.6	6.82E-12	0.9836	1.1151	0.3905	0.7123
**ADRRRR.368**	1	0.9343	0.7307	81.4	4.75E-10	0.9736	1.1261	0.3784	0.6706
**AAADDR.170**	1	0.8092	0.7924	118.4	4.13E-12	0.9797	1.1318	0.3721	0.7304
**AAADDR.87**	1	0.8092	0.7924	118.4	4.13E-12	0.9797	1.1318	0.3721	0.7304
**AAADRR.558**	1	1.0318	0.6625	60.9	8.38E-09	0.9837	1.1356	0.3679	0.6718
**AAADRR.208**	1	1.0318	0.6625	60.9	8.38E-09	0.9837	1.1356	0.3679	0.6718
**AAAADR.55**	1	1.0634	0.6416	55.5	2.16E-08	0.9699	1.1439	0.3586	0.6663
**AAAADR.56**	1	1.0634	0.6416	55.5	2.16E-08	0.9699	1.1439	0.3586	0.6663
**AAAADR.59**	1	1.0352	0.6603	60.3	9.29E-09	0.9699	1.1494	0.3524	0.6608
**AAAADR.57**	1	1.0352	0.6603	60.3	9.29E-09	0.9699	1.1494	0.3524	0.6608
**AAADRR.559**	1	0.9964	0.6853	67.5	2.79E-09	0.9796	1.1616	0.3386	0.6779
**AAADRR.209**	1	0.9964	0.6853	67.5	2.79E-09	0.9796	1.1616	0.3386	0.6779
**AAAADR.58**	1	1.0656	0.6401	55.1	2.31E-08	0.9729	1.201	0.293	0.6183
**AAAADR.60**	1	1.0656	0.6401	55.1	2.31E-08	0.9729	1.201	0.293	0.6183

Table S3: Generated phase pharmacophore models from one conformer of NAMPT inhibitors

ID	# Factors	SD	R^2	F	P	Stability	RMSE	Q^2	Pearson-R
**AADRRR.133**	1	0.8738	0.7279	66.9	1.57E-08	0.9693	0.7929	0.6174	0.8027
**AADRRR.134**	1	0.9204	0.6981	57.8	5.86E-08	0.9703	0.8451	0.5653	0.7817
**AADRRR.135**	1	0.8889	0.7184	63.8	2.43E-08	0.9605	0.8518	0.5584	0.784
**AADDRR.61**	1	0.6946	0.8281	120.4	4.76E-11	0.9604	0.8677	0.5418	0.7779
**ADRRRR.124**	1	0.8559	0.7491	71.6	1.15E-08	0.9261	0.8787	0.53	0.8016
**AAADDR.42**	1	0.9245	0.6954	57.1	6.56E-08	0.8856	0.8829	0.5256	0.7411
**AAADDR.80**	1	0.9245	0.6954	57.1	6.56E-08	0.8856	0.8829	0.5256	0.7411
**ADDRRR.34**	1	0.7977	0.782	86.1	2.08E-09	0.9684	0.8919	0.5158	0.7819
**ADRRRR.125**	1	0.9053	0.7193	61.5	4.49E-08	0.9309	0.8927	0.515	0.7893
**AADDRR.73**	1	0.6598	0.8449	136.2	1.31E-11	0.9544	0.8964	0.511	0.7714
**AADDRR.141**	1	0.6598	0.8449	136.2	1.31E-11	0.9544	0.8964	0.511	0.7714
**ADRRRR.16**	1	0.9344	0.6889	55.4	8.59E-08	0.9644	0.8984	0.5088	0.7722
**AAADRR.150**	1	0.8938	0.7153	62.8	2.79E-08	0.9692	0.899	0.5081	0.7297
**AAADRR.390**	1	0.8938	0.7153	62.8	2.79E-08	0.9692	0.899	0.5081	0.7297
**AADRRR.678**	1	0.7509	0.8069	100.3	4.79E-10	0.9769	0.9036	0.503	0.7756
**AADRRR.422**	1	0.7509	0.8069	100.3	4.79E-10	0.9769	0.9036	0.503	0.7756
**AADRRR.656**	1	1.09	0.5766	34	4.38E-06	0.9417	0.9129	0.4927	0.7247
**AAADRR.391**	1	0.9081	0.7061	60.1	4.17E-08	0.9716	0.9135	0.4921	0.731
**AAADRR.151**	1	0.9081	0.7061	60.1	4.17E-08	0.9716	0.9135	0.4921	0.731
**ADRRRR.196**	1	1.0278	0.6381	42.3	9.98E-07	0.9271	0.9248	0.4794	0.7279
**AADRRR.655**	1	0.8493	0.743	72.3	7.63E-09	0.9554	0.9279	0.476	0.7527
**ADDRRR.2**	1	0.7986	0.7727	85	1.61E-09	0.9685	0.942	0.4599	0.7765
**ADRRRR.126**	1	0.9975	0.6592	46.4	4.79E-07	0.9425	0.9497	0.4511	0.7026
**AADRRR.660**	1	0.9689	0.6655	49.7	2.16E-07	0.9421	0.9503	0.4504	0.7036
**AADRRR.677**	1	0.7216	0.8216	110.5	1.83E-10	0.9722	0.9555	0.4443	0.7624
**AADRRR.421**	1	0.7216	0.8216	110.5	1.83E-10	0.9722	0.9555	0.4443	0.7624
**ADRRRR.18**	1	0.9627	0.6698	50.7	1.84E-07	0.9475	0.9576	0.4418	0.7479
**ADDRRR.33**	1	0.8717	0.7292	67.3	1.48E-08	0.9616	0.9601	0.439	0.7584
**AADDRR.75**	1	0.8177	0.7081	50.9	4.88E-07	0.8736	0.9808	0.4369	0.6939

**AADDRR.143**	1	0.8177	0.7081	50.9	4.88E-07	0.8736	0.9808	0.4369	0.6939
**AAADRR.336**	1	0.9914	0.6498	46.4	3.88E-07	0.9679	0.9756	0.4207	0.6677
**AAADRR.106**	1	0.9914	0.6498	46.4	3.88E-07	0.9679	0.9756	0.4207	0.6677
**AADDRR.117**	1	0.8072	0.7678	82.7	2.11E-09	0.9547	0.9828	0.4121	0.7326
**ADDRRR.66**	1	0.838	0.7594	75.8	6.86E-09	0.9606	1.0029	0.3878	0.7388
**AAAADR.22**	1	0.9262	0.6715	45	9.69E-07	0.8753	1.0178	0.3854	0.64
**AADDRR.129**	1	0.9692	0.6653	49.7	2.18E-07	0.9387	1.0071	0.3827	0.6587
**ADRRRR.17**	1	1.0273	0.6239	41.5	9.61E-07	0.9583	1.015	0.3729	0.6888
**ADDRRR.1**	1	0.9378	0.6866	54.8	9.45E-08	0.9517	1.0166	0.371	0.7215
**AADDRR.63**	1	1.015	0.6328	43.1	7.08E-07	0.9509	1.0547	0.3229	0.6354
**AAADDR.78**	1	0.922	0.6971	57.5	6.13E-08	0.9556	1.056	0.3213	0.6353
**AAADDR.40**	1	0.922	0.6971	57.5	6.13E-08	0.9556	1.056	0.3213	0.6353
**AAADDR.79**	1	0.9542	0.6755	52	1.47E-07	0.9576	1.0628	0.3125	0.6059
**AAADDR.41**	1	0.9542	0.6755	52	1.47E-07	0.9576	1.0628	0.3125	0.6059
**AADDRR.65**	1	0.9092	0.7054	59.9	4.30E-08	0.9549	1.0783	0.2923	0.5888
**ADDRRR.65**	1	0.7957	0.7744	85.8	1.47E-09	0.9665	1.0814	0.2883	0.682
**ADRRRR.197**	1	1.0784	0.6016	36.2	3.24E-06	0.882	1.0824	0.2869	0.6909
**ADRRRR.198**	1	0.8969	0.7244	63.1	3.58E-08	0.9267	1.0849	0.2836	0.6845
**AAADRR.371**	1	1.0163	0.6319	42.9	7.30E-07	0.9633	1.1166	0.2411	0.6268
**AAADRR.137**	1	1.0163	0.6319	42.9	7.30E-07	0.9633	1.1166	0.2411	0.6268
**AADDRR.121**	1	0.9921	0.6492	46.3	3.95E-07	0.9493	1.1182	0.2389	0.6311
**AAADRR.372**	1	1.0389	0.6153	40	1.28E-06	0.9602	1.129	0.2242	0.6233
**AAADRR.138**	1	1.0389	0.6153	40	1.28E-06	0.9602	1.129	0.2242	0.6233
**AAADDR.81**	1	0.9254	0.6948	56.9	6.72E-08	0.9473	1.135	0.2159	0.6335
**AAADDR.43**	1	0.9254	0.6948	56.9	6.72E-08	0.9473	1.135	0.2159	0.6335
**AAADDR.77**	1	0.8934	0.7155	62.9	2.76E-08	0.9348	1.1607	0.1801	0.5647
**AAADDR.39**	1	0.8934	0.7155	62.9	2.76E-08	0.9348	1.1607	0.1801	0.5647
**AAAADR.32**	1	0.9174	0.7117	59.2	6.21E-08	0.9167	1.1682	0.1694	0.5739
**AAAADR.31**	1	0.9174	0.7117	59.2	6.21E-08	0.9167	1.1682	0.1694	0.5739
**AAAADR.20**	1	1.0405	0.6142	39.8	1.33E-06	0.9548	1.1771	0.1566	0.5416
**AAAADR.89**	1	1.0456	0.6104	39.2	1.51E-06	0.9492	1.2646	0.0266	0.4559

**AAAADR.91**	1	1.0024	0.6558	45.7	5.40E-07	0.952	1.2783	0.0055	0.4137

Table S4: Prediction result of data set through the model ('ADDRRR146')

Molecule ID	Set	pIC50	Pred Activity	Align Score	Vector Score	Volume Score	Fitness	Matched Ligand Sites
**1_03**	training	7.552842	7.144595	0.545604	0.987518	0.834509	2.239843	A(2) D(5) D(-) R(7) R(9) R(8)
**1_09**	training	7.744727	7.176042	0.545903	0.988345	0.821943	2.227943	A(2) D(5) D(-) R(6) R(8) R(7)
**1_11**	training	5.886057	6.990747	0.725794	0.96286	0.7635	2.121532	A(3) D(6) D(7) R(8) R(10) R(9)
**1_13**	training	7	6.924507	0.01396	0.999776	0.897965	2.886108	A(3) D(6) D(7) R(9) R(10) R(11)
**1_14**	training	7.267606	6.924507	0	1	1	3	A(3) D(6) D(7) R(8) R(10) R(9)
**1_15**	test	5.769551	6.87092	0.167695	0.999122	0.918467	2.489874	A(3) D(6) D(-) R(8) R(10) R(9)
**1_16**	training	5.721246	6.584759	0.088596	0.998557	0.941955	2.866683	A(2) D(5) D(6) R(7) R(9) R(8)
**1_17**	test	6.387216	6.924507	0.017743	0.999777	0.879993	2.864984	A(4) D(7) D(8) R(10) R(11) R(12)
**1_18**	test	6.39794	6.856021	0.028791	0.999722	0.869775	2.845504	A(3) D(7) D(8) R(10) R(11) R(12)
**1_19**	test	6.387216	6.924507	0.023126	0.999772	0.896813	2.877313	A(3) D(8) D(9) R(10) R(11) R(12)
**1_20**	test	6.408935	6.924507	0.021961	0.999716	0.891776	2.873191	A(3) D(6) D(7) R(9) R(10) R(11)
**1_21**	test	6.522879	6.818994	0.036862	0.932716	0.772143	2.67414	A(3) D(6) D(7) R(11) R(13) R(12)
**1_22**	training	6.356547	6.924507	0.049998	0.998948	0.851124	2.808407	A(3) D(7) D(8) R(10) R(12) R(11)
**1_23**	test	6.431798	6.88748	0.061213	0.999246	0.955107	2.903342	A(3) D(7) D(8) R(9) R(10) R(11)
**1_24**	test	6.958607	6.771745	0.072476	0.998276	0.825176	2.763056	A(3) D(7) D(8) R(11) R(13) R(12)
**1_25**	test	8.102373	6.924507	0.006101	0.999984	0.961443	2.956343	A(3) D(6) D(7) R(10) R(12) R(11)
**1_26**	test	8.21467	6.88748	0.06018	0.999222	0.866178	2.815249	A(3) D(6) D(7) R(9) R(11) R(10)
**2_07**	training	5.88941	6.706297	1.414399	0.493701	0.222008	0.593435	A(1) D(3) D(4) R(7) R(-) R(-)
**4_04**	training	8.60206	6.318416	0.731689	0.89612	0.79457	2.080949	A(2) D(5) D(6) R(8) R(7) R(9)
**4_04**	training	7.537602	6.91226	0.837125	0.812465	0.683795	1.798656	A(2) D(5) D(6) R(8) R(7) R(9)
**4_05**	training	8.619789	8.828158	0.275856	0.985991	0.777485	2.037926	A(5) D(-) D(-) R(7) R(-) R(8)
**4_05**	training	8.60206	8.127835	0.768912	0.763052	0.576227	1.560145	A(4) D(-) D(5) R(-) R(6) R(7)
**4_06**	test	8.657577	8.73069	0.272832	0.972176	0.762595	2.009618	A(5) D(-) D(-) R(10) R(-) R(9)
**4_06**	test	8.619789	8.292607	0.820019	0.83256	0.600806	1.630468	A(3) D(-) D(6) R(-) R(9) R(8)
**4_07**	training	8.657577	8.63843	0.390905	0.986092	0.703508	2.053929	A(4) D(-) D(5) R(7) R(-) R(8)
**4_07**	training	8.356547	8.828158	0.302426	0.983327	0.772457	2.026566	A(5) D(-) D(-) R(7) R(-) R(8)

**4_08**	training	8.356547	8.628035	0.409363	0.717351	0.455216	1.425447	A(5) D(-) D(-) R(10) R(-) R(9)
**4_08**	training	8.124939	8.832197	0.252051	0.976216	0.766679	2.020359	A(5) D(-) D(-) R(10) R(-) R(9)
**4_09**	training	8.267606	8.841609	0.312654	0.975709	0.669214	1.91421	A(5) D(-) D(-) R(7) R(-) R(8)
**4_09**		8.124939	8.36135	0.804201	0.81868	0.587835	1.61106	A(3) D(-) D(5) R(-) R(6) R(7)
**4_10**		8.69897	8.696378	0.270697	0.969708	0.75514	1.999974	A(4) D(-) D(-) R(8) R(-) R(9)
**4_10**		8.267606	8.055559	0.737681	0.769404	0.580305	1.584683	A(3) D(-) D(5) R(-) R(7) R(8)
**4_11**		8.769551	8.608236	0.294518	0.975767	0.765933	2.013608	A(6) D(-) D(-) R(8) R(-) R(9)
**4_11**		8.69897	8.578781	0.866735	0.780282	0.671111	1.62609	A(5) D(-) D(6) R(7) R(-) R(8)
**4_12**		8.769551	7.998053	0.747122	0.837972	0.563767	1.632483	A(4) D(-) D(7) R(-) R(9) R(10)
**4_12**		8.619789	8.803142	0.329704	0.97163	0.621564	1.859883	A(5) D(-) D(-) R(10) R(-) R(11)
**4_13**	training	9.221849	8.03879	0.744143	0.784345	0.54458	1.561007	A(4) D(-) D(5) R(-) R(8) R(9)
**4_13**	training	8.356547	8.950612	0.270023	0.984375	0.762393	2.02198	A(5) D(-) D(-) R(9) R(-) R(10)
**4_14**	training	8.721246	8.738058	0.317996	0.973784	0.65738	1.89965	A(5) D(-) D(-) R(8) R(-) R(9)
**4_14**	training	8.619789	7.705485	0.65795	0.759799	0.418158	1.447376	A(4) D(-) D(5) R(-) R(7) R(8)
**4_15**		8.443697	8.697433	0.251565	0.982382	0.687537	1.947442	A(5) D(-) D(-) R(9) R(-) R(10)
**4_16**		8.721246	7.895558	0.602787	0.788644	0.478899	1.55934	A(3) D(-) D(6) R(-) R(8) R(9)
**4_16**		7.537602	8.738058	0.300256	0.973606	0.716655	1.961355	A(5) D(-) D(-) R(9) R(-) R(10)
**4_17**	training	9.221849	8.744976	0.296494	0.9747	0.692259	1.938588	A(6) D(-) D(-) R(8) R(-) R(9)
**4_17**		8.443697	8.378781	0.811425	0.837866	0.415323	1.454345	A(5) D(-) D(6) R(-) R(7) R(8)
**5_07**	training	4.040959	4.160529	0.54321	0.508459	0.397691	1.129969	A(-) D(4) D(-) R(-) R(7) R(6)
**5_10**	training	4.468521	4.119721	0.567669	0.487763	0.387477	1.093005	A(-) D(3) D(-) R(-) R(6) R(5)
**5_12**	training	4.420216	4.119721	0.537048	0.534431	0.346435	1.106175	A(-) D(4) D(-) R(-) R(6) R(7)
**5_14**	training	4.30103	4.313892	0.391205	0.538075	0.411463	1.205805	A(-) D(3) D(-) R(-) R(6) R(5)
**5_25**		4.886057	4.315708	0.429211	0.513842	0.418974	1.181839	A(-) D(3) D(-) R(-) R(4) R(5)
**5_26**		4.886057	4.262372	0.419646	0.578107	0.442008	1.271017	A(-) D(3) D(-) R(-) R(5) R(6)
**5_27**		6	4.356516	0.454009	0.631747	0.55125	1.426974	A(-) D(3) D(-) R(-) R(4) R(5)
**5_28**		6	4.356516	0.445283	0.600984	0.566273	1.413038	A(-) D(3) D(-) R(-) R(5) R(6)
**5_30**		6.376751	5.519407	0.562754	0.65304	0.507787	1.468043	A(4) D(5) D(-) R(-) R(8) R(9)

**5_31**		4.987163	4.880881	0.468932	0.763742	0.576228	1.680321	A(3) D(5) D(-) R(-) R(7) R(8)
**5_33**		4.886057	4.716774	0.88989	0.731754	0.471748	1.366869	A(2) D(5) D(-) R(-) R(8) R(9)
**5_34**		5.481486	4.934438	0.839034	0.779214	0.435453	1.402726	A(2) D(5) D(-) R(-) R(7) R(8)
**5_35**		5.30103	4.778155	0.601245	0.759431	0.546384	1.598219	A(3) D(5) D(-) R(-) R(7) R(8)
**5_36**	training	6.21467	5.543082	0.863416	0.314632	0.271018	0.76196	A(-) D(4) D(5) R(-) R(7) R(6)
**5_37**		4.60206	5.255393	0.552985	0.732199	0.533635	1.576702	A(3) D(4) D(-) R(-) R(6) R(7)
**5_39**		6.677781	6.120437	0.882266	0.876506	0.504627	1.548246	A(4) D(6) D(-) R(-) R(9) R(8)
**5_40**		5.823909	5.15224	0.569424	0.750922	0.537088	1.592707	A(3) D(4) D(-) R(-) R(7) R(8)
**5_41**		4.39794	4.997052	1.025999	0.800134	0.422191	1.31641	A(3) D(4) D(-) R(-) R(7) R(8)
**5_42**	training	4.080922	4.952339	0.576195	0.700947	0.566108	1.569175	A(3) D(5) D(-) R(-) R(8) R(9)
**5_43**	training	7.853872	6.173773	0.884742	0.865091	0.504644	1.535634	A(4) D(6) D(-) R(-) R(10) R(9)
**5_44**		6.19382	5.152141	0.506904	0.766841	0.538476	1.632789	A(3) D(4) D(-) R(-) R(7) R(8)
**5_45**		5.508638	5.03354	0.872095	0.764557	0.375721	1.312365	A(3) D(4) D(-) R(-) R(7) R(8)
**5_46**		4.207608	4.952339	0.531307	0.72924	0.567513	1.615561	A(3) D(5) D(-) R(-) R(8) R(9)
**5_47**		7.721246	4.823274	0.838156	0.780951	0.479251	1.448681	A(3) D(5) D(-) R(-) R(8) R(9)
**5_48**		6.537602	5.328384	0.579431	0.737629	0.507797	1.546307	A(3) D(4) D(-) R(-) R(7) R(8)
**5_49**	training	4.09691	4.973438	1.000306	0.827526	0.420712	1.355725	A(3) D(4) D(-) R(-) R(7) R(8)
**5_50**		4.443697	4.75551	0.621695	0.730584	0.559707	1.57456	A(3) D(5) D(-) R(-) R(8) R(9)
**5_51**		7.721246	6.485688	0.756041	0.907021	0.489326	1.623067	A(4) D(6) D(-) R(-) R(10) R(9)
**5_52**		5.823909	6.343656	1.320474	0.720756	0.350928	0.987375	A(-) D(4) D(5) R(6) R(7) R(8)
**5_53**		5.040959	4.971996	0.475504	0.328193	0.399269	1.065638	A(-) D(5) D(-) R(6) R(7) R(8)
**5_54**		5.468521	4.971996	0.482394	0.295366	0.395912	1.027149	A(-) D(5) D(-) R(6) R(7) R(8)
**5_55**	training	4.585027	4.989699	0.454683	0.270257	0.389036	1.004278	A(-) D(4) D(-) R(5) R(6) R(7)
**5_56**		5.387216	5.587285	0.906734	0.362124	0.358458	0.875618	A(-) D(4) D(-) R(5) R(6) R(7)
**5_57**		4.677781	4.971996	0.468825	0.367755	0.397769	1.10591	A(-) D(4) D(-) R(5) R(6) R(7)
**5_58**		4.677781	5.191974	0.47923	0.339455	0.390065	1.066453	A(-) D(3) D(-) R(4) R(5) R(6)
**5_61**		5.853872	4.971996	0.474079	0.307552	0.39149	1.037691	A(-) D(4) D(-) R(6) R(7) R(8)
**5_62**		5.431798	5.043035	0.473906	0.328828	0.335322	1.002857	A(-) D(4) D(-) R(6) R(7) R(8)

**5_63**		7.823909	7.22003	0.863598	0.533765	0.273838	0.983824	A(-) D(-) D(5) R(8) R(7) R(10)
**5_64**		5.21467	5.052148	0.491775	0.303069	0.389378	1.025141	A(-) D(4) D(-) R(7) R(8) R(9)
**5_65**	training	4.853872	5.38707	0.843547	0.368992	0.308955	0.863843	A(-) D(3) D(-) R(5) R(6) R(7)
